# Intramedullary Nail Fixation with Autologous Bone Marrow Transplantation in an Incomplete Atypical Femoral Fracture Patient: Use of Bone Marrow Extracted from the Hollow Reamer

**DOI:** 10.1155/2020/2878651

**Published:** 2020-08-25

**Authors:** Ryutaro Iwasaki, Masaaki Sakamoto, Tomoyuki Rokkaku, Hitoshi Watanabe, Toshiyuki Yamada, Takeshi Yamaguchi, Yasushi Wako, Hitoshi Kubosawa, Seiji Ohtori

**Affiliations:** ^1^Department of Orthopaedic Surgery, Graduate School of Medicine, Chiba University, Japan; ^2^Department of Orthopaedic Surgery, Chiba Municipal Aoba Hospital, Japan; ^3^Department of Pathology, Chiba Municipal Aoba Hospital, Japan

## Abstract

The present report describes an incomplete atypical femoral fracture (AFF) patient who underwent simultaneous autogenous bone transplantation to the resected fracture region during intramedullary nail fixation. A 73-year-old female with a history of multiple myeloma had been undergoing treatment with intravenous drip injections of Zoledronic Acid. She was introduced to our department due to the left lateral thigh pain, with no trauma incidence. An anteroposterior radiograph showed a transverse thin fracture line with localized periosteal and endosteal thickening, which is compatible with subtrochanteric incomplete AFF. A biochemical investigation revealed the existence of severely suppressed bone turnover. She underwent intramedullary nail fixation for fear of a complete fracture. After the fixation, the cortical bone at the fracture region was excised as a wedge-shaped block, and bone marrow extracted from the hollow reamer was simultaneously transplanted to the resected fracture region. Histological examination showed few bone formation features at the fracture line in the excised lateral cortical bone. At 7 months after surgery, radiographs demonstrated complete bone repair, and no clinical problems were observed two years postoperatively. To the best of our knowledge, this is the first report in which autogenous bone marrow transplantation, noninvasive to the iliac crest, was performed in an incomplete AFF patient. We believe that this low invasive procedure can be a useful technique for AFF treatment.

## 1. Introduction

In 2005, Odvina et al. [1] reported that long-term alendronate therapy could cause severely suppressed bone turnover (SSBT), resulting in delayed healing of nonspinal fractures. Since then, SSBT after prolonged use of bisphosphonates (BPs) has been considered to be one of the causes of low-energy femoral fractures, called “atypical femoral fracture” (AFF). Delayed healing has long been a clinical problem in AFF cases under SSBT [2, 3]. Moreover, recent histological studies clarified few bone formation features at the thin fracture lines of incomplete AFF cases [4]. The present report shows a minimally invasive procedure, which could facilitate bone union in incomplete AFF. The clinical course and pathological results of the patient are presented with a review of the related literature.

## 2. Case Presentation

**Figure 1:**
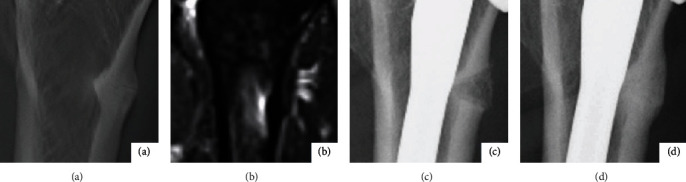
Anteroposterior radiograph showing a transverse thin fracture line with localized periosteal and endosteal thickening at the left subtrochanteric lateral cortex (a). Coronal STIR image showing focal cortical thickening with minimal edema of the adjacent bone marrow (b). Anteroposterior radiographs showing fracture region just after surgery (c) and complete bone repair at 7 months after surgery (d).

A 73-year-old female with a history of multiple myeloma had been undergoing treatment with intravenous drip injections of Zoledronic Acid over three years in our hospital. She was introduced to our department due to an increase of weight-bearing pain in her left lateral thigh, with no trauma incidence. Anteroposterior radiographs and magnetic resonance images were compatible with subtrochanteric incomplete AFF (Figures 1(a) and 1(b)) [2, 5, 6]. A biochemical investigation revealed a serum calcium of 9.2 mg/dl, phosphorus of 4.1 mg/dl, and 25-hydroxyvitamin D of 62 ng/ml, indicating vitamin D sufficiency. Tartrate-resistant acid phosphatase 5b was 245 mU/dl and bone-specific alkaline phosphatase level was 6.1 *μ*g/l, clarifying the existence of SSBT [7]. Dual-energy X-ray absorptiometry at lumbar spine and hip revealed T scores of −1.0, −1.3, and Z scores of 1.1, 1.0. Although teriparatide was not used due to a medical contraindication, Zoledronic Acid treatment was discontinued based on the judgment of an internist. Since severe loading pain continued for approximately 3 months, surgical procedures were considered to be necessary for fear of a complete fracture.

**Figure 2:**
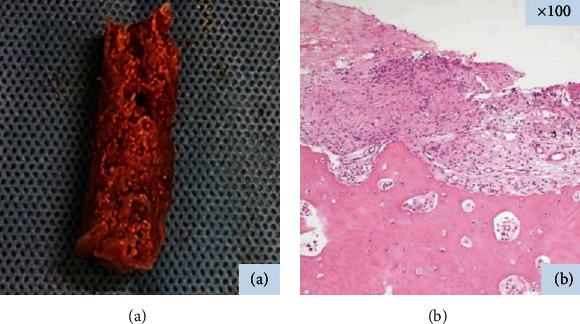
Intraoperative photograph showing the cylindrical bone marrow extracted from the hollow reamer (a). Histologic image at the fracture line in the excised lateral cortical bone. Photomicrograph showing few osteoblasts and little woven bone (hematoxylin-eosin, original magnification x 100) (b).

After intramedullary nail fixation, the skin incision was extended 2 cm distally from the blade insertion. The cortical bone at the fracture region was excised as a wedge-shaped block to reach the medullary cavity with a chisel. Bone marrow remains inside the hollow reamer were extracted for use as an autologous bone graft (Figure 2(a)), which was transplanted to the resected fracture region.

Histological examination showed few bone formation features at the fracture line in the excised cortical bone (Figure 2(b)). No atypical cells were pathologically identified in the bone marrow pieces inside the hollow reamer. Anteroposterior radiograph just after surgery demonstrated bone marrow transplanted to the resected fracture region (Figure 1(c)). The postoperative course was uneventful, and pain decreased promptly. At 4 months after surgery, radiographs demonstrated apparent cortical bone remodeling, and complete bone repair was observed at 7 months after surgery (Figure 1(d)). No clinical problems were observed two years postoperatively.

## 3. Discussion

**Figure 3:**
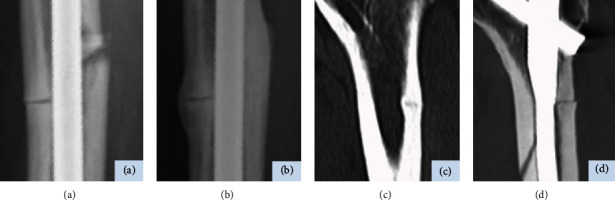
Images of the first reference case (a, b) and the second reference case (c, d). Anteroposterior radiographs showing fracture region just after surgery (a) and incomplete bone remodeling in the lateral cortex at 2 years after surgery (b). Reconstructed coronal computed tomography (CT) scans showing a thin fracture line in the lateral cortex before surgery (c) and a complete fracture at approximately 3 years after surgery (d).

Since Odvina et al. [1] reported patients sustaining low-energy fractures after prolonged BPs use, there have been many reports regarding AFF including etiology, pathophysiology, and the treatment outcome [8, 9]. Delayed healing is one of the minor features in the American Society of Bone and Mineral Research task force, and as a matter of fact, we also experienced 2 AFF cases with unusual bone healing. The first reference case is a 74-year-old female who underwent intramedullary nailing for complete AFF. Although complete healing was observed throughout most of the whole cortex bone circumference at 2 years after surgery, the fracture surface in the lateral cortex, despite contact, showed little bone remodeling radiographically (Figures 3(a) and 3(b)). The second reference case is a 76-year-old female who underwent intramedullary nailing for incomplete AFF. Approximately 3 years later, a fall accident led to a complete fracture, which originated from the same prior fracture line in despite thick nailing (Figures 3(c) and 3(d)). Based on these clinical experiences, we focused on the thin fracture lines in the lateral cortex.

Koh et al. [10] named the thin fracture line “dreaded black line”, which was considered as an indicator for the progression of complete fractures and as a state of chronic nonunion for microcrack accumulation under SSBT with continued BPs. In a histological study, Schilcher et al. [4] showed that the thin fracture line contained amorphous, nonmineralized materials. Histological findings in the present case also showed few bone formation features at the thin fracture line in the lateral cortex, which was compatible with previous reports.

Cortical bone repair is considered to be necessary for a complete bone union [11]. Several recent reports have described additional surgical techniques for the fracture region in AFF patients. Bögl et al. [12] reported incomplete AFF patients who underwent reamed intramedullary nail fixation with excision of the fracture region. This technique brought continuity over the defect with dense bone at a median of 17 months, which indicates remodeling activity in the bone adjacent to the thin fracture line. Lovy et al. [13] reported that the use of bone marrow aspirate concentrate from the iliac crest reduced time to bone union in AFF. Several basic studies have reported the significant role of myeloid cells in fracture healing; therefore, it can be assumed that bone marrow transplantation is certainly effective for bone union [14, 15]. The iliac crest is currently the most common donor site for obtaining the autogenous bone graft, which can be an invasive procedure. Consequently, bone marrow extracted from the hollow reamer was used to prevent invasive autogenous bone collection, resulting in smooth bone remodeling. We believe that the good result was brought by both excisions of the incomplete fracture region with poor remodeling activity and bone marrow transplantation to the resected region.

To the best of our knowledge, this is the first report in which autogenous bone marrow transplantation, noninvasive to the iliac crest, was simultaneously performed during intramedullary nail fixation in incomplete AFF patients. Furthermore, the procedure brought about good clinical results. Although further research is necessary to verify the effectiveness and limitation of additional surgical techniques in the fracture region, our procedure can be considered to be a simple method in the acceleration of fracture healing in AFF.

## 4. Conclusion

A subtrochanteric incomplete AFF patient, who underwent autogenous bone marrow transplantation to the resected fracture region simultaneously with intramedullary nail fixation, is presented. Bone marrow extracted from the hollow reamer was used as an autogenous bone graft, resulting in a satisfactory bone union. We believe that this low invasive procedure can be a useful technique for AFF treatment.
